# An 18S rRNA Workflow for Characterizing Protists in Sewage, with a Focus on Zoonotic Trichomonads

**DOI:** 10.1007/s00248-017-0996-9

**Published:** 2017-05-24

**Authors:** Julia M. Maritz, Krysta H. Rogers, Tara M. Rock, Nicole Liu, Susan Joseph, Kirkwood M. Land, Jane M. Carlton

**Affiliations:** 1 Center for Genomics and Systems Biology, Department of Biology, New York University, New York, NY 10003 USA; 2 Wildlife Investigations Laboratory, California Department of Fish and Wildlife, Rancho Cordova, CA 95670 USA; 3 Department of Biological Sciences, University of the Pacific, Stockton, CA 95211 USA

**Keywords:** Sewage, Protist, Zoonoses, Trichomonad, 18S rRNA amplicon sequencing, Environmental sequencing

## Abstract

Microbial eukaryotes (protists) are important components of terrestrial and aquatic environments, as well as animal and human microbiomes. Their relationships with metazoa range from mutualistic to parasitic and zoonotic (i.e., transmissible between humans and animals). Despite their ecological importance, our knowledge of protists in urban environments lags behind that of bacteria, largely due to a lack of experimentally validated high-throughput protocols that produce accurate estimates of protist diversity while minimizing non-protist DNA representation. We optimized protocols for detecting zoonotic protists in raw sewage samples, with a focus on trichomonad taxa. First, we investigated the utility of two commonly used variable regions of the 18S rRNA marker gene, V4 and V9, by amplifying and Sanger sequencing 23 different eukaryotic species, including 16 protist species such as *Cryptosporidium parvum*, *Giardia intestinalis*, *Toxoplasma gondii*, and species of trichomonad. Next, we optimized wet-lab methods for sample processing and Illumina sequencing of both regions from raw sewage collected from a private apartment building in New York City. Our results show that both regions are effective at identifying several zoonotic protists that may be present in sewage. A combination of small extractions (1 mL volumes) performed on the same day as sample collection, and the incorporation of a vertebrate blocking primer, is ideal to detect protist taxa of interest and combat the effects of metazoan DNA. We expect that the robust, standardized methods presented in our workflow will be applicable to investigations of protists in other environmental samples, and will help facilitate large-scale investigations of protistan diversity.

**Electronic supplementary material:**

The online version of this article (doi:10.1007/s00248-017-0996-9) contains supplementary material, which is available to authorized users.

## Introduction

Microbes are the most abundant and diverse organisms in the biosphere, detected in almost every ecosystem, *e.g* *.*, in and on humans [1], pets [2], the built environment [3], soil [4], and the ocean [5]. Microbial communities are composite populations of thousands of microorganisms whose collective presence and relative abundance reflect conditions of the surrounding environment. During the last decade, high-throughput sequencing technologies have revolutionized our understanding of these complex systems and their implications for human health. For example, sewage contains microorganisms from human and animal waste as well as from groundwater, soil, and other environments [6]. Sewage is also a major reservoir for human and animal pathogens that are known to vary based on the host source of the waste, exposure to which may pose severe threats to environmental and public health [7]. Additionally, recent studies have shown that sewage accurately reflects the microbial composition of human stool, and it may be possible to identify host-specific microbes from sewage that could serve as indicators of fecal pollution sources [8–10].

The majority of these studies, however, have primarily aimed to characterize the prokaryotic diversity of these communities. In contrast, the protistan component of these ecosystems remains relatively unexplored, largely due to a lack of standard marker genes and reference databases. Protists are important components of terrestrial and aquatic environments, where they are integral constituents of trophic chains and nutrient cycles [11]. This includes human-made ecosystems such as wastewater treatment facilities, where they play roles in the purification process [12]. Human and animal microbiomes are also home to various protist species whose relationships with their hosts vary from parasitic to mutualistic [13]. Zoonotic (i.e., transmissible between humans, domesticated animals, and wildlife) protists such as species of *Giardia*, *Blastocystis*, *Cryptosporidium*, and a variety of trichomonads, *e.g* *.*, *Tritrichomonas fetus*, are common parasites of humans, livestock, other domestic animals and wildlife, contributing to significant host morbidity and mortality [14]. Exposure to, and risks from, these diseases are compounded in urban environments where contact between host and reservoir species is increased. Despite their ecological and economic importance, little is known about the diversity, incidence, or emergence of zoonotic parasites—or protists in general—in urban environments.

Broad surveys of eukaryotes in sewage have been explored on a very limited basis but their composition has been shown to reflect contributions from various animal, human, and environmental sources [15]. Microbial surveys of raw sewage should reflect community patterns and present an ideal system to monitor zoonotic parasites. For example, recent studies suggest that trichomonads (anaerobic, flagellated protists belonging to the large and diverse groups Trichomonadea and Tritrichomonadea of phylum Parabasalia [16]) are crossing host boundaries [17]. The recent isolation of new species of avian trichomonads responsible for epidemic outbreaks in California with high genetic similarity to human trichomonads highlights the possibility of zoonotic transfer from humans to birds and/or vice versa [18]. With the co-habitation of birds and humans in many urban and suburban areas, bird feces containing these parasites could contaminate human water sources and present a public health threat. The ability to distinguish between and monitor these parasites in sewage may provide insight regarding their distribution and potential transmission routes.

Understanding the prevalence and distribution of trichomonads and other zoonotic protists in sewage samples requires accurate methods for detection and identification. Current methods for high-throughput eukaryotic diversity studies rely on sequencing variable regions of the small ribosomal subunit (18S rRNA gene); however, no single region is universally accepted for environmental marker gene studies [19]. Several previous *in silico* and high-throughput studies have been conducted which suggest the V4 and V9 regions are the most variable, and best suited for microbial eukaryote studies [20–22]. While the utility of the V4 and V9 regions has been discussed in previous literature [23–26], no studies have conducted a direct comparison between primer sets using Illumina technology, nor have many studies investigated their resolution at deeper taxonomic levels, such as between closely related species or strains of zoonotic taxa. Additionally, parameters of sample collection and processing, DNA extraction, and sequencing protocols need to be evaluated, which are particularly important in studies of eukaryotes due to the potential masking effects of host DNA. Some studies have measured the impact of masking effects on the recovery of bacterial communities from stool samples, but such potential biases have yet to be assessed for protists or sewage samples [27].

Here, we describe an optimized workflow for the detection and analysis of protists in sewage samples, with a focus on zoonotic and trichomonad taxa, based on high-throughput amplicon sequencing of existing 18S rRNA markers. First, using Sanger sequencing and *in silico* testing methods, we compared the abilities of two regions (V4 and V9) to distinguish between a variety of human and animal-infectious protist taxa likely to be present in sewage, including *Cryptosporidium parvum*, *Giardia intestinalis*, *Toxoplasma gondii*, and several species of trichomonad. We then developed optimized protocols for sample processing and Illumina sequencing of these regions for raw sewage collected from a private apartment building in New York City. Our study also provides effective methods for high-throughput library construction and deep sequencing for generating high-quality sequencing data. These data provide a standard protocol for the detection of zoonotic protists in sewage, and pave the way forward for further investigation in sewage and other environmental samples.

## Methods

### Protist and Vertebrate Samples

Genomic DNA was eluted from *C*. *parvum*, *T*. *gondii*, *Blastocystis hominis*, *G*. *intestinalis*, rat, chicken, dog, and horse, following instructions specified by each provider. Genomic DNA was extracted directly from frozen stabilates of *Monotrichomonas carabina*, *Ditrichomonas honigbergii*, *Trichomitus batrachorum*, *Monocercomonas colubrorum*, all *Trichomonas gallinae* stabilates from the American Type Culture Collection (https://www.atcc.org), and both *Tetratrichomonas gallinarum* stabilates, using DNAZol and following the manufacturer’s instructions. Other protist species were obtained as genomic DNA, including *Entamoeba invadens* and *Entamoeba histolytica* (from Dr. Daniel Eichinger, New York University School of Medicine); *Dientamoeba fragilis* (from Dr. Graham Clark, London School of Hygiene and Tropical Medicine); and *Trichomonas tenax* (from Dr. Andrew Brittingham, Des Moines University, Iowa). The scientific name, strain, geographical location, and reference of all known organisms used in this study are shown in Online Resource 1.

For trichomonad species, wild caught birds (including Band-tailed pigeons, Eurasian collared doves, Mourning doves, Ring-necked pheasants, White-winged doves) were sampled between June 2014 and January 2015 in the state of California, USA. Sterile cotton-tipped applicator swabs moistened with sterile saline were used to collect oral swabs from each bird. After collection, swabs were used to inoculate InPouch TF (BioMed Diagnosticts), a transport and culture device designed for the detection and growth of *T*. *foetus* parasites that has also been shown to work for avian isolates [28]. Inoculated InPouches were incubated anaerobically at 37 °C and examined by microscopy for the presence of protozoa. Positive InPouches, or those containing motile trichomonads as determined by microscopy, were then subcultured into Peptone Yeast Extract Maltose (TYM) medium. Axenic cultures were obtained using subsequent culturing over several weeks in TYM as well as treatment with anti-fungal drugs as described previously in [18, 29]. Isolates still growing after 1 week of antibiotic treatment were then subjected to several rounds of serial dilution to generate parasites for DNA extraction. Genomic DNA was extracted from clonal isolates using a DNAEasy Blood & Tissue extraction kit (QIAGEN, catalog #69504).

### Sewage Sample Collection and DNA Extraction

Two 50 mL samples of raw sewage were collected from the aerated feed tank in the basement of a private apartment building in New York City in July and September of 2014, and transported in secure containers to New York University. Sewage samples were handled under Bio Safety Level 2 conditions in a laminar flow hood, with the handler wearing personal protective clothing and goggles. Fresh DNA extractions were performed using 11 1 mL aliquots per sample on the day of sample collection with the PowerSoil DNA Isolation kit (MOBIO, catalog #12888) following manufacturer’s instructions. DNA from 10 of the 11 aliquots was concentrated post-extraction using a SpeedVac (Savant) and subsequently pooled to represent DNA from 10 mL of sewage. This resulted in four samples per time point, two representing 1 mL of sewage and two pooled 10 mL samples. DNA extractions were performed on an additional 10 mL of sewage from each of the July samples after they were stored for 1 week at −20 °C as previously described. No extractions were performed on frozen sewage from the September samples. DNA concentration was quantified using the Qubit dsDNA HS Assay kit (Invitrogen, catalog #Q32851).

### 18S rRNA Amplification for Sanger Sequencing

Primers TAReuk454FWD1 (5′-CCAGCASCYGCGGTAATTCC-3′), TAReukREV3 (5′-ACTTTCGTTCTTGATYRA-3′) [24] were used to target the V4 region of the 18S SSU rRNA gene, and universal primers 1391f (5′-GTACACACCGCCCGTC-3′ [30]) and EukBr (5′-TGATCCTTCTGCAGGTTCACCTAC-3′ [31]) were used to target the V9 variable region. Fragments were amplified from total genomic DNA using either Phusion High-Fidelity DNA Polymerase (NEB, catalog #M0530S) or Phusion High-Fidelity PCR Master Mix (Thermo Scientific, catalog # F-531S) in a 50 μL reaction volume, with 1–5 μL input DNA (depending on concentration), PCR grade water, and a final primer concentration of 0.5 mM. Amplification conditions for the V4 region were 98 °C for 30 s, 30 cycles of 98 °C for 10 s, 49 °C for 30 s, 72 °C for 30 s, and a final step of 72 °C for 10 min. Reaction conditions for the V9 region were 98 °C for 30 s, 30 cycles of 98 °C for 10 s, 62 °C for 30 s, 72 °C for 30 s, and a final step of 72 °C for 10 min. PCR products were visualized on a 1% agarose gel, successful amplifications were purified using a 1.8X ratio of Agencourt AMPure XP beads (Beckman Coulter, catalog #A63880) and sent to Genewiz for Sanger sequencing. For templates that did not amplify initially, 1.5 μl of DMSO per reaction was added and the PCR was repeated.

### 18S rRNA Amplification for Illumina Sequencing

Environmental DNA extracted from sewage samples was prepared for Illumina sequencing targeting the V4 region, and also the V9 region with and without the addition of the mammal blocking primer. The V4 region was amplified with Illumina primer constructs containing the TAReuk454FWD1 and TAReukREV3 primers; no blocking primer is available for this region. Library synthesis and amplification were performed in triplicate using Phusion High-Fidelity PCR Master Mix (Thermo Scientific, catalog #F-531S), a 20 μL reaction volume, and a two-step PCR amplification strategy as described in [24]: 98 °C for 30 s, 10 cycles of 98 °C for 10 s, 53 °C for 30 s, 72 °C for 30 s; and then 25 cycles of 98 °C for 10 s, 48 °C for 30 s, 72 °C for 30 s, and ending at 72 °C for 10 min.

The V9 fragment of the 18S rRNA gene was amplified using Illumina primer constructs containing the universal primers 1391f-EukBr [23], and the mammal blocking primer designed as described in [32]. Library synthesis and amplification using 5 μL of input DNA was done in triplicate following the Earth Microbiome protocol [33, 34]. All V9 region primers, including the blocking primer, and protocols for amplification and sequencing are available on the EMP website (http://www.earthmicrobiome.org/emp-standard-protocols/18s/).

We included several negative controls, including extraction blanks for DNA purification experiments, and no-template blanks as negative controls in PCR reactions. No bands were visible on agarose gels after amplification, and no DNA was able to be quantified using the Qubit dsDNA HS Assay kit, and thus these control samples were excluded from downstream Illumina sequencing. After amplification, triplicate PCR reactions were pooled and purified with a 1.8X ratio of AMPure XP beads. The size distribution of purified libraries was determined using the 2100 Bioanalyzer or 2200 TapeStation (Agilent Technologies), and quantified via qPCR using the Library Quantification Kit—Illumina/LightCycler® 480 (KAPA Biosystems, Roche® LightCycler 480).

Quantified libraries were individually normalized to 4 nM based on qPCR and Bioanalyzer values and equal amounts of each 4 nM dilution were pooled. Eight samples were multiplexed per Illumina sequencing run. MiSeq preparation and sequencing was performed based on the manufacturer’s and Earth Microbiome protocols [35] using the following parameters. Pools for the V4 region were sequenced at a final concentration of 12 pM with a 10% PhiX control spike-in using an Illumina MiSeq 500 cycle V3 kit and 2 × 300 run configuration. Pools for the V9 region were sequenced at a final concentration of 10 pM with a 6% PhiX control spike-in using an Illumina MiSeq 300 cycle V2 reagent kit with a 2 × 100 run configuration.

### Analysis of Sanger Sequences

Sanger sequences for the V4 and V9 regions were processed in Geneious 7.1.7 [36]. Sequences were trimmed of poor quality areas using an error probability limit of 0.05, assembled using the de novo option and any remaining primer sequences were removed from the consensus sequence. We used the 18S rRNA SSU sequences in GenBank for *D*. *fragilis Bi/PA* (U37461), *G*. *intestinalis* Portland-1 (M54878), and *E*. *invadens* (AF149905) in all analyses, since these samples did not produce high-quality sequences for both regions.

Multiple sequence alignments were created using the MUSCLE [37] alignment option within Geneious using default parameters, and are available in FASTA format for the V4 data in Online Resource 2 and in Online Resource 3 for the V9 data. MrBayes 3.2.2 [38, 39] was used for phylogenetic analysis of trichomonad sequences and run using 10^6^ generations, a sampling frequency of 500, and the GTR substitution model with gamma-distributed rate variation and a proportion of invariable sites. The resulting trees were visualized using FigTree (v1.4.2) [40].

All Sanger sequences from the V4 and V9 regions used in this study were de-replicated and clustered into OTUs at both 97 and 98% identity cutoff values using USEARCH v8.0.1 [41]. GenBank sequences were used where high-quality Sanger sequences were not obtained. Taxonomy was assigned to representative OTU sequences using BLAST [42] within QIIME against the full QIIME compatible SILVA database [43] version 111 (http://www.arb-silva.de/download/archive/qiime/) and an in-house curated version (see below) with an e-value of 1e-15.

### Data Analysis of Illumina Sequences

Processing of Illumina sequencing reads from the sewage data was performed at the same time for both regions. Paired-end reads were joined within the QIIME 1.8.0 pipeline [44] using fastq-join [45] with a minimum overlap of 10 bp and allowing a 15% error rate in the overlapping area. Joined reads were de-multiplexed and quality filtered (split_libraries_fastq.py, -q 19 –r 5 –p 0.70), and any reverse primers detected were truncated (truncate_reverse_primer.py). Due to the poor quality of read 2 for the V4 region (average quality value, Q30, of 30.9%), read 1 was trimmed to 250 bp using Trimmomatic [46], then de-multiplexed using the parameters above. De-multiplexed reads for both regions were subject to *de novo* chimera checking, removal of singletons, and clustered into OTUs at 98% identity following the UPARSE pipeline (USEARCH v8.0.1, [47]). Taxonomy was assigned to representative OTU sequences using BLAST within QIIME, first against our curated SILVA database (see below), and subsequently with the QIIME formatted SILVA 111 database clustered at 99% identity. OTUs with no significant hits (<90% identity) after both rounds of taxonomic assignment were labeled as “Unidentified.” Both datasets were filtered to remove non-18S sequences (bacterial and archaeal OTUs) and low abundance OTUs making up <0.0005% of reads in the total dataset as recommended for Illumina sequencing data [48].

Eukaryotic OTU tables were rarefied to 600,000 sequences per sample with 10 repetitions prior to alpha diversity analysis. Alpha diversity analyses (Phylogenetic Diversity metric, Shannon Index) were carried out using the QIIME pipeline on the 10 rarefied OTU tables. For metrics requiring a phylogenetic tree, representative OTU sequences were aligned to the curated database using PyNAST [49] at an identity threshold of 70%, a minimum length of 70 bp, and filtered to remove gaps present in >98% of sequences, followed by construction of phylogenetic trees using the default settings of FastTree [50]. Results were plotted using the ggplot2 [51] R package and statistically compared using either the Wilcox Rank Sum or Kruskal-Wallis tests in R. Univariate tests for differentially abundant taxa with respect to extraction volume and primers used was performed on sum-normalized phylum level taxonomic summaries of non-rarefied eukaryotic OTU tables using LEfSe [52] with default settings (Alpha = 0.05, LDA > 2, All-against-all or strict comparison). Samples were grouped into classes and analyzed based on extraction volume, primers used, or both depending on the amplicon region.

Eukaryotic OTU tables were further filtered to represent only protist taxa by excluding all metazoan, fungi, and plant sequences, and low abundance protist OTUs were removed as described above. The resulting OTU tables were rarefied stepwise from 60,000 to 600,000 sequences with 10 repetitions per level and rarefaction curves of the Phylogenetic Diversity metric were generated for each region.

### SILVA Database Curation

The SILVA version 111 QIIME formatted database was downloaded (ftp://ftp.microbio.me/pub/QIIME_nonstandard_referencedb/Silva_111.tgz) and the 60,584 eukaryotic sequences from the full, unaligned files without ambiguous bases (Silva_111_full_unaligned_no_ambig.fasta) were filtered to remove all “unidentified,” “uncultured,” “clone,” and “environmental sample” sequences. The remaining eukaryotic sequences were further curated with a focus on protist taxa. This included removing and or re-annotating mislabeled sequences and adding taxonomic placeholders representing super-groups, subphyla, *etc*. Sequences from protists of interest missing from the original database were downloaded from GenBank and added to the curated version. A list of the GenBank accession numbers and taxonomic strings of the sequences added can be found in Online Resource 4. A template alignment for this database was created by aligning it to the eukaryotic representative sequence file clustered at 99% identity (99_Silva_111_rep_set_euk_aligned.fasta), provided in the original QIIME formatted database, using PyNAST and an identity threshold of 60%.

### Data Accessibility

Sequence data have been deposited in National Center for Biotechnology Information public databases. Sanger sequences have GenBank accession numbers KU939249, KU939251-KU939275, KU939280-KU939294, KU939300-KU939316, KU939319, KU939320, KU939326, KU939328-KU939352, KU939357-KU939371, and KU939377-KU939394. Raw Illumina sequencing data is archived in the Short Read Archive (SRA) under BioProject PRJNA315104. Mapping files for each 18S region, including SRA IDs, the curated SILVA database, and the eukaryotic abundance filtered OTU tables used for diversity analyses in this study can be downloaded from Figshare [10.6084/m9.figshare.3114850].

### Results

**Fig. 1:**
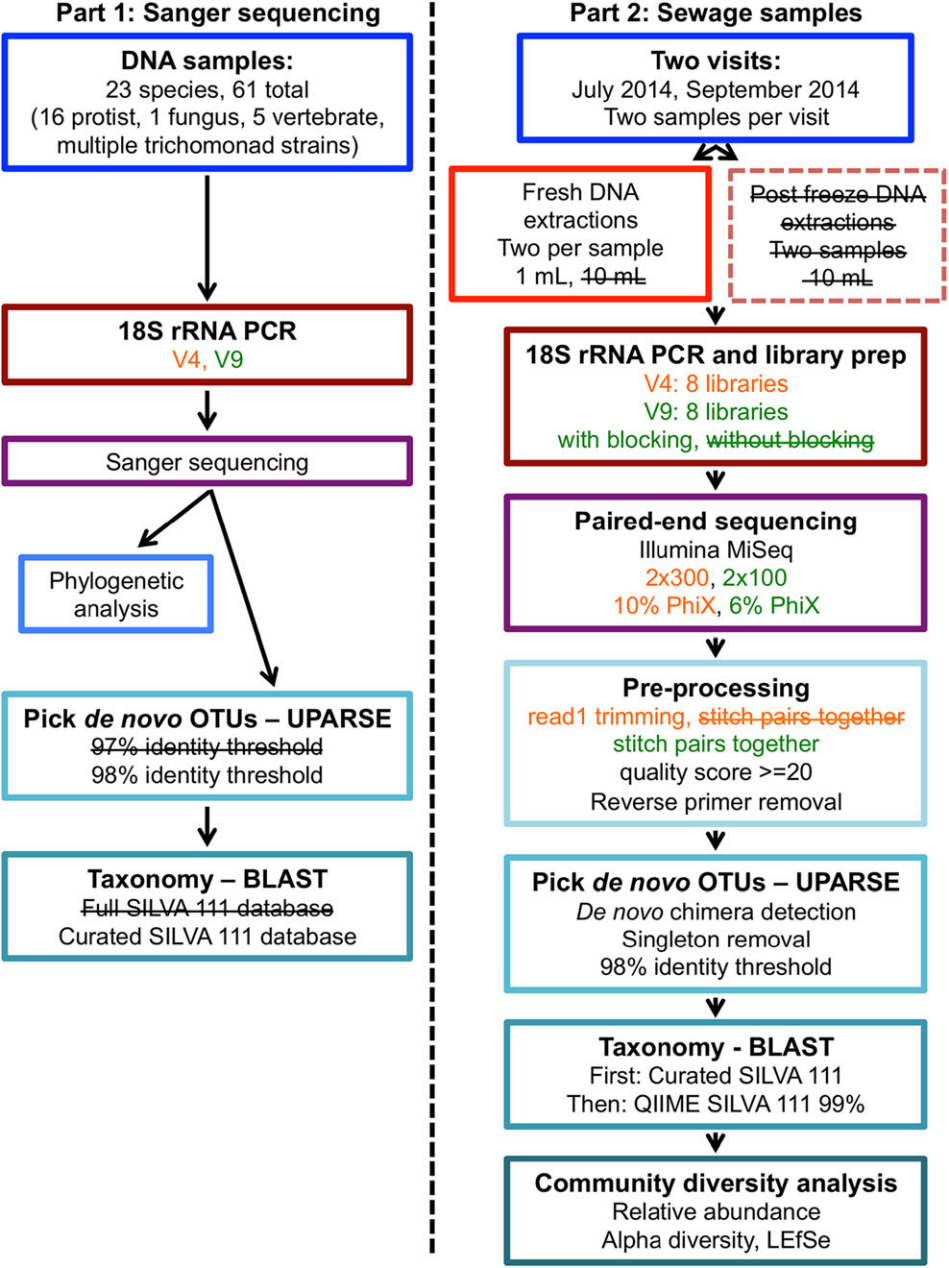
Workflow employed for testing and optimization of protocols to detect microbial eukaryotes in raw sewage. Part 1 was used to evaluate two variable regions of the 18S rRNA gene, V4 and V9, and determine best practices for zoonotic protists. Part 2 was used to optimize the methods in Part 1 and develop experimental methods for Illumina sequencing of raw sewage samples. Methods that were tested but determined suboptimal are indicated in *boxes with dotted lines* and/or *crossed out text*

We used two approaches for our method optimization (illustrated in Fig. 1). In part one, we tested the abilities of two variable regions of the 18S rRNA gene, V4 and V9, to detect and distinguish between a variety of human and animal infectious protist taxa likely to be present in sewage using Sanger sequencing. We also used these sequences as a mock community to evaluate parameters for protist community analysis. In part two, we developed experimental protocols for processing and sequencing of raw sewage samples, and further evaluated the use of V4 and V9 regions for protist diversity studies and techniques.

### Evaluation of Published 18S rRNA Primers and Reference Databases

We first tested the ability of two of the most variable regions of the 18S rRNA gene, V4 and V9, which are widely used in studies of eukaryotic microbial biodiversity, to distinguish between a variety of protist species and distinguish protist DNA from vertebrate DNA likely to be present in sewage. Primer pairs TAReuk454FWD1 and TAReukREV3, and 1391f and EukBr were used to amplify the 18S rRNA gene of 23 different eukaryotic species including 16 protists, 1 fungus, and 5 vertebrates. We also included multiple isolates of several species of trichomonads, totaling 61 DNA samples in all (Online Resource 1). Amplicons ranging from 270 to 387 bp in length for the V4 region and 96–134 bp for the V9 region were subsequently sent for Sanger sequencing. High-quality sequences were obtained for all samples using V4 primers except *G*. *intestinalis*. High-quality sequences were obtained for all but two samples for the V9 region (*D*. *fragilis* and *E*. *invadens*).

**Fig. 2:**
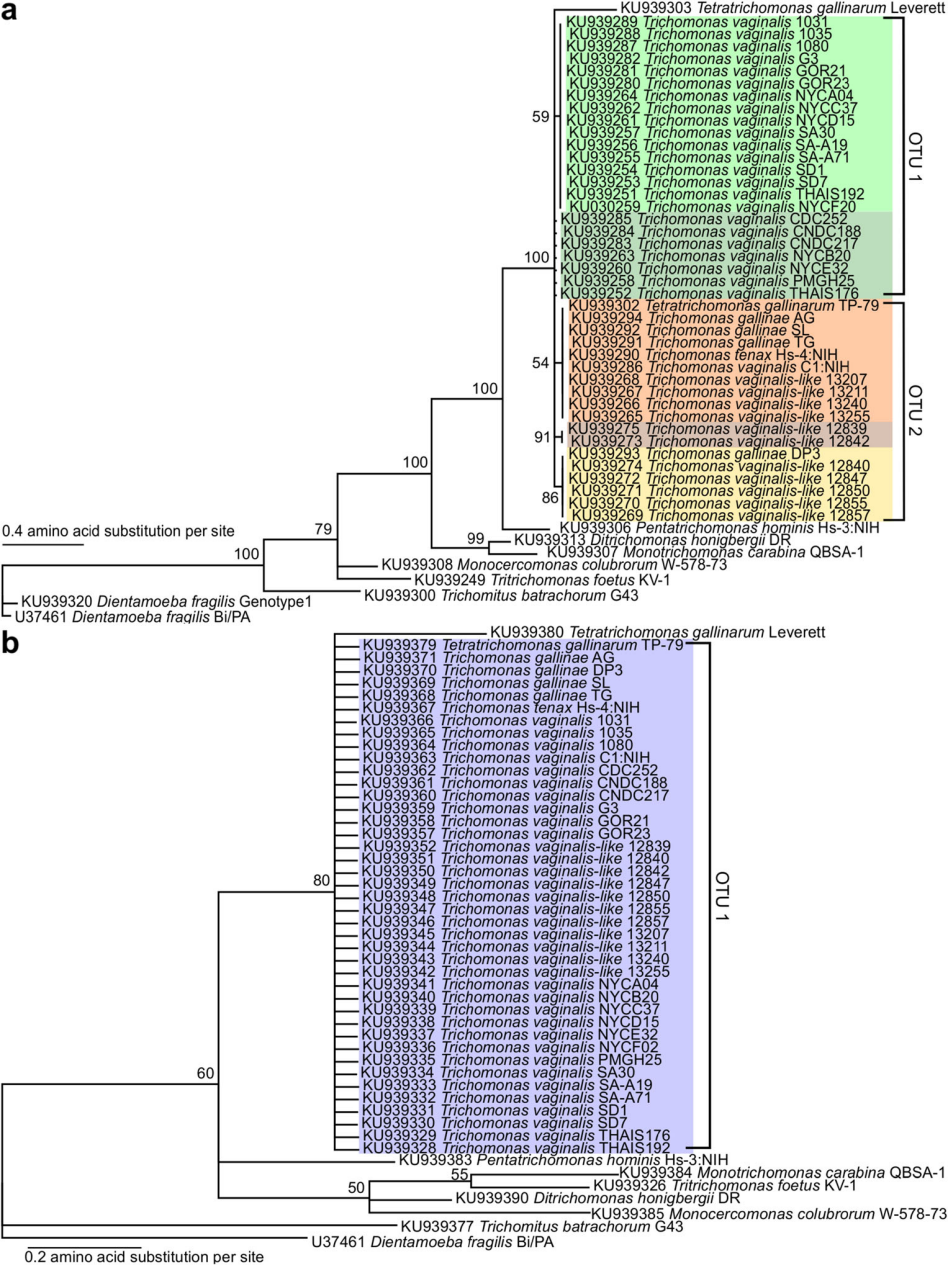
Phylogenetic tree of trichomonad Sanger sequences from **a** V4 region and **b** V9 region. Numbers at nodes correspond to Bayesian posterior probabilities. GenBank accession numbers are shown at the left for each taxon. Colored taxa are identical to each other and brackets indicate OTU membership and corresponding ID number from the supplementary material after clustering at 98% identity

V4 region amplicon sequences had a pairwise identity (PI) of 69.7% (Online Resource 2). All four mammalian species had a PI >99% relative to one other, but were distinguishable from the chicken DNA sample. Phylogenetic analysis of the trichomonad V4 sequences is shown in Fig. 2a, where they formed five different groups of identical sequences. Two of the five are composed exclusively of *Trichomonas vaginalis* sequences (Fig. 2a**,** green), and two others are almost entirely *T*. *vaginalis*-like sequences (Fig. 2a**,** brown and yellow). The fifth group shows the most diversity, with a mixed representation of *T*. *vaginalis*, *T*. *tenax*, *T*. *gallinae*, and *T*. *gallinarum* sequences (Fig. 2a, orange). All five of these clusters are >97% identical to each other (the standard cutoff used to differentiate OTUs in prokaryotes). V9 region amplicon sequences had an average pairwise identity of 79.4% (Online Resource 3) and a similar pattern as the V4 between the vertebrate species. Phylogenetic analysis of the trichomonad V9 sequences (Fig. 2b) showed less variation within the trichomonad clade compared to the V4 data. Four trichomonad species, *T*. *vaginalis* (all 24 strains), *T*. *vaginalis*-like (all 11 strains), *T*. *tenax*, *T*. *gallinae* (all four strains), and one strain of *T*. *gallinarum* (TP-79), had identical sequences for this region. The other strain of *T*. *gallinarum* (Leverett) was distinguishable from the other trichomonads with a PI of 96% to the trichomonad group.

Next, we used our Sanger sequences as input for a mock community analysis to evaluate the phylogenetic resolution and taxonomic accuracy of these primers in a microbial community setting. OTU clustering of the 63 Sanger sequences from the V4 region, representing 23 different species, returned 19 OTUs at 97% identity and 20 OTUs at 98% identity. At 97% identity, the 42 sequences from the 4 different trichomonad species clustered into 2 OTUs, 1 representing 41/42 sequences and the other representing *T*. *gallinarum* Leverett (Online Resource 5). The four mammalian species clustered into one OTU. At 98% identity, the trichomonad sequences clustered into three distinct OTUs, one representing 23/24 *T*. *vaginalis* sequences, the second representing all *T*. *gallinae* (4 sequences) and *T*. *vaginalis*-like (11 sequences), *T*. *tenax*, one *T*. *gallinarum* (TP-79), and one *T. vaginalis* (C1:NIH) (Fig. 2a, Online Resource 5). The third trichomonad OTU represents *T*. *gallinarum* Leverett. The 62 V9 Sanger sequences were clustered into 18 OTUs at both the 97% and 98% identity cut off values. The sequence composition of these OTUs was identical using both methods and was in accord with the phylogenetic trees. The pattern of sequence clustering was similar to that of the V4 at 97% identity, with all trichomonad sequences clustered together in one OTU with the exception of *T*. *gallinarum* Leverett, and the four species of mammals represented by one OTU (Fig. 2b, Online Resource 6).

We then assigned taxonomy to these OTUs using the full SILVA 111 QIIME formatted database and compared the taxonomy of the recovered hit to that of the known query. Primers for the V4 region showed higher taxonomic accuracy than the V9, matching the query ID for 14/20 OTUs at the species level and 15/20 at the genus level. The V9 correctly identified 8/18 OTUs at the species level and 13/18 at the genus level. The unrecovered OTUs were identified as either other species in the same genus or closely related taxa (Online Resource 5 and Online Resource 6). Reference sequences for three taxa (*M*. *carabina*, *E*. *histolytica*, *E*. *invadens*) were not present in the database used for identification.

We curated the existing SILVA 111 QIIME reference database by adding sequences for these missing taxa and others of interest (see Materials and Methods and Online Resource 4). This resulted in a total of 46,094 eukaryotic sequences with curated taxonomy. We then re-assigned taxonomy to the OTUs obtained from our Sanger sequences. After database curation, all 20 V4 OTUs were correctly identified at the genus level, and all except *B*. *hominis* were correctly identified at the species level (Online Resource 5). Of the V9 OTUs, 11 out of 18 were correctly recovered to the species level, including *M*. *carabina* and *E*. *invadens* and 16 out of 18 to the genus level (Online Resource 6). The OTU representing the four mammal sequences was identified as a closely related species, but not one of the four member taxa.

### Development of Experimental Protocols for Raw Sewage

We collected samples of raw sewage from a private apartment complex in New York City in order to develop sample processing protocols and further test the utility of the V4 and V9 18S rRNA regions for protist diversity studies. This green apartment building has its own wastewater and rainwater recycling system where treated water is reused for toilets, laundry facilities, and garden irrigation. This “closed” system represents an ideal site for methods testing because the sewage there is mostly from human residents, with little environmental input, which allows for evaluation of the background of metazoan DNA and provides a control for protists that are human based that may be obscured by other taxa in more open systems. Two 50 mL samples were collected at two time points, July and September 2014, and DNA extracted (Fig. 1).

First, we determined optimal preprocessing conditions for sewage samples considering the constraints of same-day extraction, technician fatigue for processing multiple samples, and potential bias that can be introduced as a result of suboptimal collection and storage conditions. We compared the yield of DNA extracted on the day of collection from 10 mL of sewage to that extracted from 1 mL, for both July samples. DNA extracted from 10 mL of sewage gave higher yields than from 1 mL (Table 1). We also tested stability of DNA extracted from sewage that was processed immediately, compared to an additional 10 mL DNA extraction from each July sample that had been frozen for a week at −20 °C. The amount of DNA extracted from the frozen sample was dramatically lower than from the fresh sample, with an average loss of 20.2 ng/uL (Table 1). Based on these results, we confined the rest of our study to extractions performed on the day of sewage collection.

**Table 1:** DNA yield from July sewage samples with and without storage at −20 °C for 1 week

Sample ID	Day extracted	Input volume	DNA yield (ng/μL)
S005_1_mL	Day of collection	1 mL	2.9
S005_10_mL	Day of collection	10 mL	35.0
S005_10_mL_frozen	After 7 days at −20 °C	10 mL	5.81
S006_1_mL	Day of collection	1 mL	3.56
S006_10_mL	Day of collection	10 mL	21.0
S006_10_mL_frozen	After 7 days at −20 °C	10 mL	9.8

Next, we compared the effects of extraction volume and use of a vertebrate blocking primer (V9 region only) on the diversity of sewage. We collected an additional two samples in September and extracted DNA from 1 and 10 mL of sewage each on the day of collection. Illumina sequencing libraries were generated from total DNA from fresh extractions for the V4 region (8 libraries total), and for the V9 region with and without the blocking primer (16 libraries total), and run on a MiSeq to generate paired-end (PE) reads. Sequencing of the V4 libraries resulted in 18,269,491 raw PE reads. However, the quality of the sequencing reads was low; read 1 quality scores decreased significantly in the last 50 bp, and read 2 had an average Q30 of 30.9% (a successful V3 2 × 300 run should have a Q30 >70%). As a result, we were unable to join the majority of read pairs (Table 2). To obtain the highest quality and deepest sequence coverage for downstream analysis, we compared the amount of usable reads returned post de-multiplexing and quality filtering for joined read pairs, read 1 only, and read 1 trimmed to remove the last 50 poor quality bases. Un-joined read 1, trimmed to 250 bp, returned the greatest amount of high-quality (average *Q* score >19) reads for downstream analysis (Table 3). All further V4 analyses were conducted on this dataset.

**Table 2:** Reads returned after joining and/or de-multiplexing the V4 sequencing data

Analysis step	Joined	Read 1 only	Read 1 trimmed
Raw sequences or pairs	18,269,491	18,269,491	18,269,491
Joined pairs of reads	53,526	NA	NA
De-multiplexed reads	42,257	6,906,307	7,027,716

**Table 3:** Summary of Illumina sequence data for each 18S region

Analysis step	V4 region (read 1)	V9 region
Raw sequences or pairs	18,269,491	29,400,737
Joined pairs of reads	NA	22,864,953
De-multiplexed reads	7,027,716	21,395,270
Clustered reads (OTUs)	6,477,688 (2270)	21,205,088 (8827)
Eukaryotic reads (OTUs)	6,477,688 (2270)	18,794,581 (7195)
Filtered eukaryotic reads (OTUs)	6,464,532 (824)	18,664,054 (1444)
Average sequences per sample	808,066	1,196,152

De-multiplexed reads were next clustered into OTUs at a 98% identity threshold and filtered to remove low abundance OTUs. This resulted in a total of 6,464,532 eukaryotic sequences and 824 OTUs after data processing and filtering for the V4 region (Table 3). For the V9 region, a total of 29,400,737 PE reads were generated from 2 MiSeq runs. The quality of these reads was much higher compared to the V4 sequences and we were able to join the majority of read pairs resulting in 18,664,054 eukaryotic sequences and 1444 OTUs after filtering (Table 3).

### Analysis of Illumina Sequence Data from Raw Sewage Samples

**Fig. 3:**
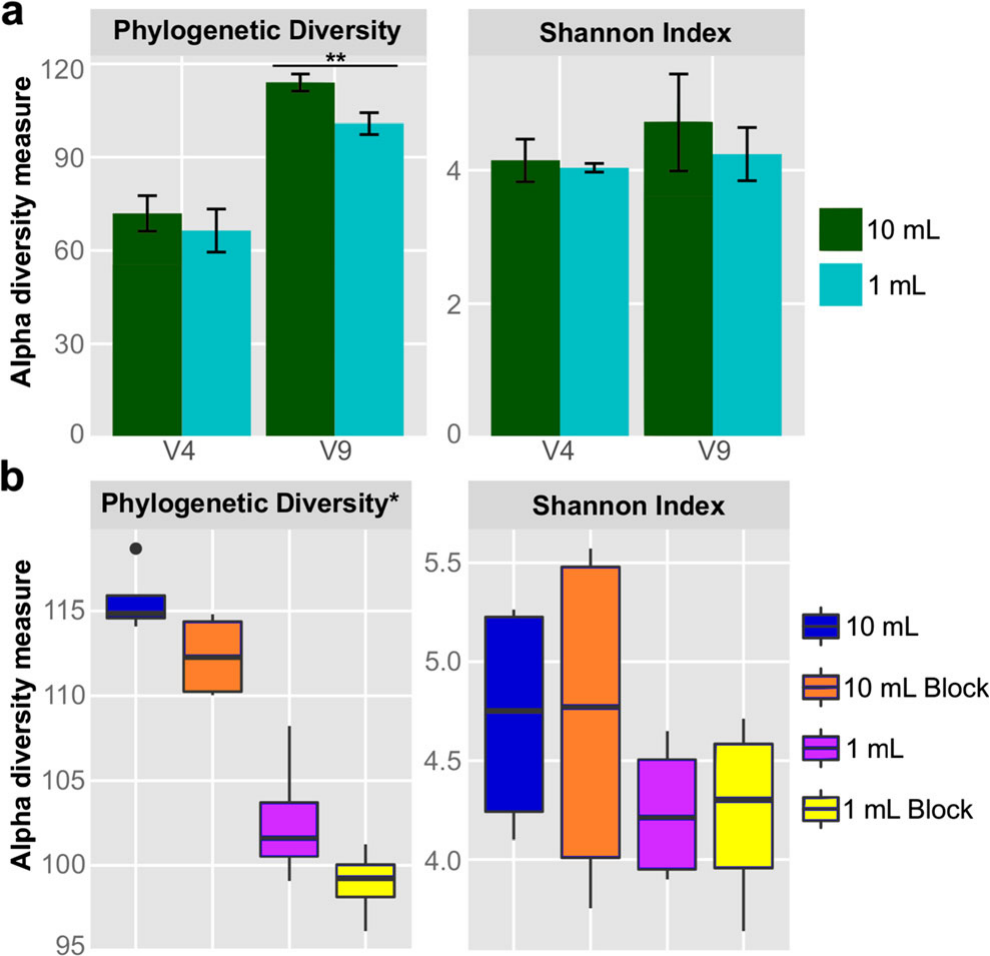
Alpha diversity analysis for sewage 18S communities using the phylogenetic diversity metric and Shannon Index. All analyses were calculated from replicate OTU tables sampled at a depth of 600,000 sequences. **a** Alpha diversity of the different extraction volumes for the V4 and V9 regions. Values shown represent the measurements from all 1 and 10 mL (fresh samples only) extraction volumes by region. *Error bars* represent ± one standard deviation; *double asterisks* indicates *p* value <0.001. **b** Alpha diversity of sewage samples based on extraction volume and blocking primer use for the V9 region. *Asterisk* indicates *p* value <0.01

We used QIIME to calculate alpha diversity, a measure of the mean species diversity, for the data from the V4 and V9 libraries. The alpha diversity of the V4 region was always lower than that of the V9 region, regardless of the initial volume of sewage (Fig. 3a). Alpha diversity of both 18S regions was lower for 1 mL samples than 10 mL samples (Fig. 3a), although the only significant pairwise comparison was the V9 phylogenetic diversity metric (Wilcox test, *p* < 0.001). For samples with the blocking primer (V9 region only), the higher extraction volume continued to show higher alpha diversity than the 1 mL extractions; however, the addition of the blocking primer reduced the phylogenetic diversity of those samples (Fig. 3b). Differences in extraction volume and primer set had small effects on alpha diversity independently, but together produced significant differences for the V9 region (Kruskal-Wallis test, *p* < 0.01).

**Fig. 4:**
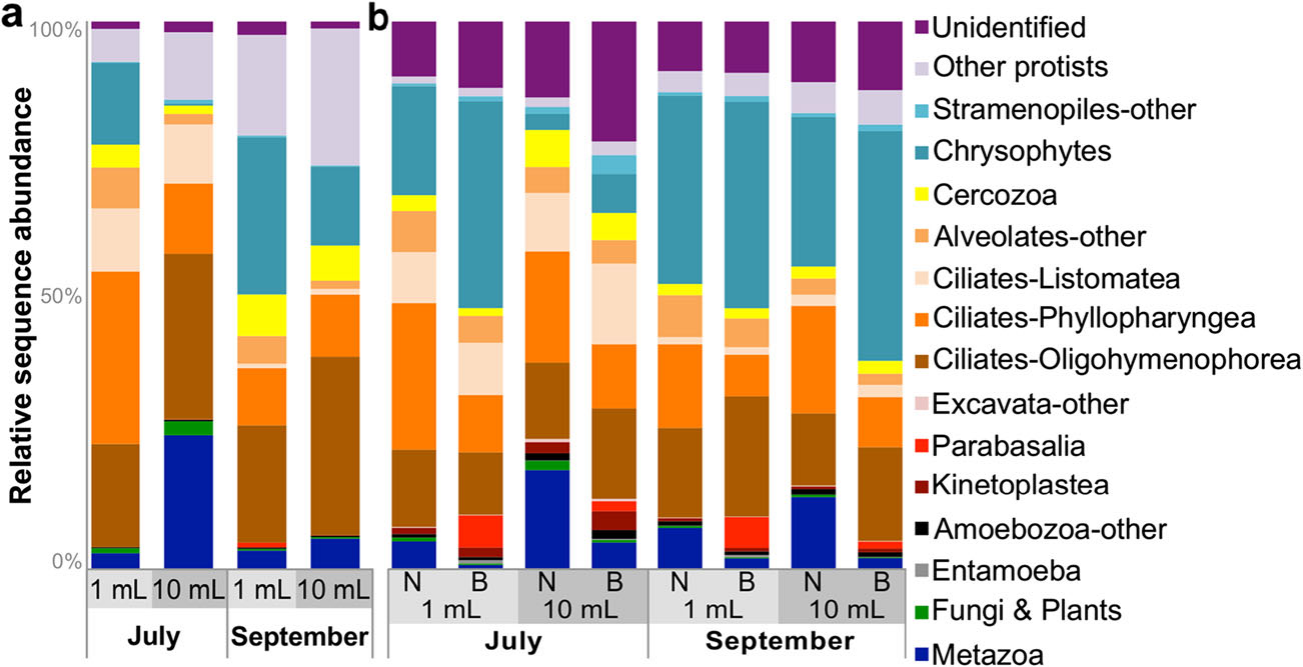
Relative sequence abundances of eukaryotic taxa across experimental conditions for each region. Each *bar* represents the average of two replicates per condition per time point. All data shown were from fresh extractions. **a** V4 region results from the 1 and 10 mL extractions. **b** V9 region results from the 1 and 10 mL extractions (with (B) and without (N) the blocking primer)

We calculated the relative sequence abundance of taxonomic assignments for all samples and 18S regions (Fig. 4). Ciliates were the dominant group (30–70% of all taxa) in sewage irrespective of extraction volume, blocking primer used, or region amplified. In particular, species of Oligohymenophorea and Phyllopharyngea, free-living bacteriovorous and common freshwater protist inhabitants of sewage were most abundant [12, 15, 53, 54]. Other highly abundant taxa included bacteriovorous flagellates belonging to the Chrysophyceae, which are typically found in fresh water and soil (1–40%). Free-living (*D*. *honigbergii*) and gut associated (*Pentatrichomonas hominis*, *D*. *fragilis*) trichomonads, along with a species originally isolated from avian sources (*Trichomonas* sp.) were detected by both regions at low abundances (≤1–5%) in sewage. We also detected low amounts of fungi, and protist species (*Entamoeba* and *Blastocystis*) that are characteristic of the mammalian gut [55]. Higher abundances of these protists were observed in the V9 samples amplified using the blocking primer than in either the V9 samples amplified without it or the V4 region. Other protist taxa of interest (*Cryptosporidium* and *Toxoplasma*) were present at very low levels (<1%) and we did not detect *Giardia* in any of our Illumina sequencing data. The V4 region showed more variable distributions of taxa between the conditions and an increase in uncultured environmental taxa included with “other protists”; in contrast, the V9 had a higher proportion of unidentified OTUs.

The amount of metazoan DNA detected by both regions ranged from <1 to 25% and consisted mostly of invertebrate taxa. The most abundant metazoan groups identified were rotifers, nematodes, and annelids, and much lower levels (<1% in all samples and regions) of human and other mammal DNA were detected. In data amplified with the V4 region, the highest amount of metazoan DNA was present in the 10 mL extraction volumes (6–25%; Fig. 4a). High levels of metazoan DNA were also found in the V9 samples amplified without the blocking primer, with the highest amounts present in the 10 mL sample size (13–18%; Fig. 4b). The V4 region showed a much larger range of metazoan DNA abundances and identified a wider variety of metazoan taxa than the V9 region. The lowest overall amounts of metazoan DNA was present in the 1 mL volumes amplified with the blocking primer (<1–2%; Fig. 4b).

**Fig. 5:**
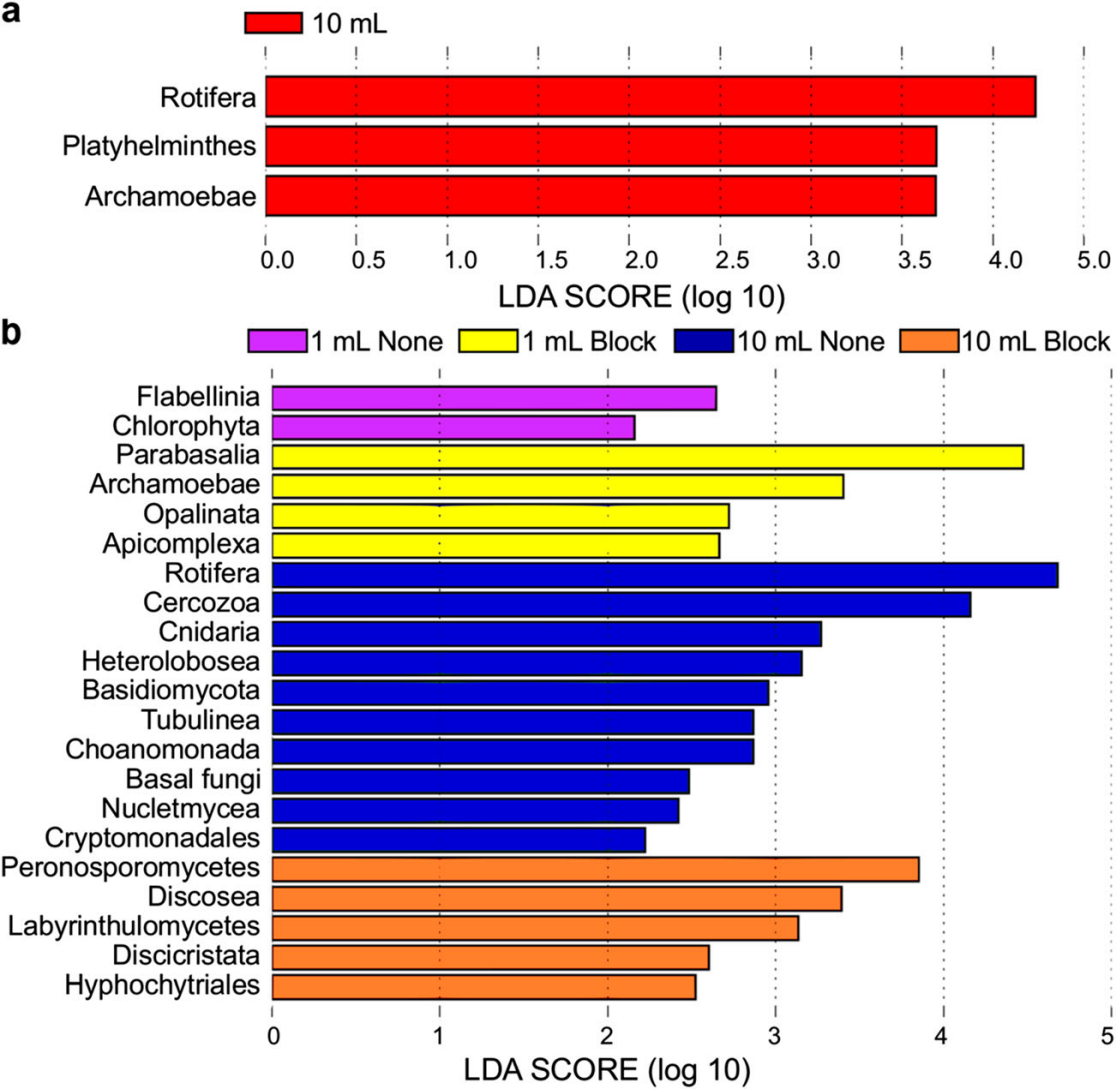
LEfSe analysis of 18S communities to determine biomarker taxa across sample groupings for each region. All data shown were from fresh extractions. LDA scores for taxa identified as differentially abundant between **a** 10 and 1 mL for the V4 region and **b** 1 mL, 1 mL blocking, 10 mL, and 10 mL blocking samples for the V9 region. Taxa are ranked according to their effect size (LDA score) and associated with the group with the highest median

Linear discriminant analysis (LDA) effect size (LEfSe) analysis was used to identify biomarkers (enriched or differentially abundant taxa) associated with sample groupings (Fig. 5). Samples from a 10 mL extraction volume were broadly enriched for metazoan and fungal (V9 only) phyla. For the V4 region, these included Rotifera and Platyhelminthes (Fig. 5a), and Rotifera, Cinardia, Choanomonada, and a variety of fungi for the V9 data (Fig. 5b). No clades were consistently present in all 1 mL samples amplified using V4 region primers. Samples from a 1 mL extraction volume amplified with V9 region primers were enriched for protist phyla and those amplified using the blocking primer were enriched for protists of interest, such as Parabasalia (trichomonads), Archamoebae (*Entamoeba*), Opalinata (*Blastocystis*), and Apicomplexa (Fig. 5b). Phylogenetic diversity-based rarefaction curves of protist-only OTU tables approach an asymptote around 350,000 protist sequences for the V4 region and around 400,000 for the V9 region (Online Resource 7). However, after removing host and other non-target sequences, some samples from a 10 mL extraction volume did not contain enough sequences to adequately capture the sample’s protist diversity.

## Discussion

In this study, we present a method for detecting microbial eukaryotes in raw sewage, while combating the possible effects of metazoan DNA. To our knowledge, this is the first study to optimize methods for investigation of zoonotic protists in raw sewage samples. We first tested the ability of primers from two 18S rRNA variable regions, the V4 and V9, to amplify a representative sample of known animal-infecting protists such *C*. *parvum*, *G*. *intestinalis*, *T*. *gondii*, and several species of trichomonads. Sanger sequencing demonstrated that both amplicons could be used to distinguish between a variety of protist taxa that may be present in sewage samples. The V4 region showed more sequence variability and higher taxonomic accuracy than the V9 region, which is consistent with results of other studies and could suggest that it is more variable at finer scales of taxonomic resolution [19, 24]. High levels of sequence similarity (above the 97% level commonly used to differentiate OTUs in bacteria) were observed in both regions, particularly between mammalian and trichomonad taxa. Due to this similarity, neither region was able to produce OTUs resolving all individual species in our mock analysis.

As predicted, the V4 region was better for discriminating between *Trichomonas* and *Tritrichomonas* genera than the V9 region. Although the V4 region could not be used to further differentiate isolates among the group of *Trichomonas* species in the mock analysis, this region could be useful to rapidly distinguish trichomonads that infect non-human mammals, such as dogs and cats, from those that infect avian species. The close grouping of avian *T*. *vaginalis*-like and human *T*. *vaginalis* isolates confirms a possible zoonotic potential for these parasites [17]. Monitoring their presence in sewage and other environmental matrices could prove valuable to public health.

We further evaluated the use of the V4 and V9 regions for protist diversity studies by Illumina sequencing raw sewage samples. The V9 region produced a larger number of OTUs at every stage of analysis than the V4 region, even though these represent highly conserved three-domain primers that also amplified low numbers of Eubacteria and Archaea. This conflicts with the data obtained from our analysis of Sanger sequences. Fewer lineages (Phylogenetic Diversity) and lower Shannon Index values were also observed for the V4 data compared to V9 data. This suggests that the primers for the V9 region may have a broader recognition spectrum than the V4 [19, 23]. This may, however, also be a product of the significantly lower quality and quantity of sequencing reads produced for the V4 region. Previous studies also observed high sequencing error rates in the V4 region due to significant length polymorphism and secondary structure [24, 56, 57]. Additionally, this dataset only used read 1 from the V4 Illumina data as we were unable to join enough read pairs for use in downstream analysis. Performance of the V4 region may improve if full-length sequences can be obtained.

The V4 and V9 regions produced broadly similar protist taxonomic profiles across sewage samples. Each primer set also showed differences in the distribution and overall diversity of taxa detected. In particular, the V9 probe detected much higher levels of Chrysophyte, and unidentified sequences, while the V4 region detected more Cercozoan and uncultured environmental sequences. The V9 region also detected higher levels of protist taxa of interest, particularly trichomonads, than the V4 region. In each case, most of the variation between regions was due to the presence of taxa not recovered by the other primer set. This is likely due to a combination of factors including bias in different primers sets that cause preferential amplification of particular protist taxa, the degree of variability present in each SSU region between particular taxa, and unbalanced representation of sequences in existing databases. Overall, our results confirm previous studies that suggest the V9 region provides a more comprehensive overview across all eukaryotes, and the V4 can better discriminate between some closely related taxa.

Previous studies showed that employing multiple primer sets increases the number of protist species detected in environmental samples [23, 24, 58]. The results of our study lead us to concur and recommend the use of both the V4 and V9 markers to provide a more accurate picture of eukaryotic diversity and better taxonomic resolution of zoonotic protists than either one on its own. Additionally, using both markers facilitates direct comparison with other datasets, as many studies use at least one of these primer sets, and provides a better level of standardization for future studies.

We conducted several experiments to determine optimal methods for processing and sequencing of sewage samples. Our study found that freezing sewage samples dramatically decreased the DNA yield, even from large volumes (Table 1), highlighting the importance of extracting DNA on the day of collection. Comparison of extraction volume and use of a vertebrate blocking primer (V9 region only) in sewage showed that samples with larger extraction volumes have higher overall levels of diversity. These samples, however, were also enriched for metazoan taxa and required deeper sequencing depths to adequately capture the protist diversity present. Surprisingly, the majority of metazoan DNA detected in this study represented invertebrate or nematode taxa, not humans or other mammals, and was successfully reduced by the blocking primer, even though it is designed for vertebrate species. This suggests that the V4 and V9 primers may be inherently biased against recovering vertebrate 18S DNA, and the recovery of vertebrate DNA in raw sewage is not as much of a concern as might be expected. Although samples with 1 mL extraction volumes had reduced eukaryotic diversity, these samples showed higher overall abundances of protist taxa. When combined with the blocking primer, the 1 mL samples were enriched for trichomonads and other taxa of interest, compared to the 10 mL samples. Thus, for our protist-focused research, a combination of small 1 mL extraction volume and incorporating the blocking primer proved most ideal for detecting protist taxa of interest and combating the effects of metazoan DNA.

To expand the applicability of our method, we developed a reliable and optimized protocol for amplicon PCR, library construction, and sequencing of the 18S V4 and the V9 regions on the Illumina MiSeq. Our protocol takes advantage of several experimental techniques including bead-based PCR cleanup and size-based quantification of individual samples to produce high-quality sequencing libraries. This, combined with dilution and pooling of individual libraries for sequencing, resulted in more even sequencing coverage across samples, and reduced the amount of data lost in downstream processing. We also observed that a lower loading concentration, adjusted based on the final concentration of the library pool, increased the overall data output and made it possible to reduce the amount of PhiX control needed. We used as little as ~6% (V9 region, V2 kit) PhiX to produce reads of comparable or higher quality to normal Illumina runs. This strategy maximizes the amount and quality of data generated with less space dedicated to sequencing PhiX and increases the depth of coverage.

Sequencing coverage is an important factor in microbiome studies. Previous research showed that broad sampling with shallow coverage (as few as 100 sequences per sample) is sufficient to adequately capture the diversity present and reveal broad ecological patterns in prokaryotic communities [33, 35, 59]. However, studies of microbial eukaryotes are prone to contamination from non-target taxa such as host or food DNA and in some cases primers that also amplify non-18S rRNA targets. In general, deeper sequencing of these communities is required to provide sufficient coverage after removing non-target OTUs and to capture rare taxa or more subtle ecological effects. For this reason, we employed a deep-sequencing strategy, multiplexing only eight samples per Illumina run (≥800,000 average sequences per sample, Table 3). This strategy more than adequately captured the protist diversity present in sewage as rarefaction curves leveled off around 350,000–400,000 protist sequences. In future runs, we have the flexibility for some cost-cutting modifications of the protocol, such as multiplexing more samples, although we recommend generating a minimum of 200,000 raw sequences per sewage sample to account for host and other non-target sequences. Many of these steps can be adapted for use with automated robotic technology to use in large-scale studies.

OTU clustering was carried out in this study using a sequence similarity cutoff of 98%, as some previous studies suggest that 97% is too conservative for estimating diversity in microbial eukaryotes [24]. Our mock analysis of Sanger sequencing data supported this idea. More species-level OTUs were captured when clustering was performed at 98% similarity for the V4 region. The taxonomic assignment of these reads, however, is still only trustworthy at the genus level. We maximized this accuracy by using a curated SILVA database and adopting a two-step process to assign taxonomy to sewage OTUs. We chose this approach because several target taxa were missing from current databases. After database curation, we correctly identified all V4 OTUs and 16 out of 18 V9 OTUs to the genus level in our mock analysis. Our curated reference database only contains eukaryotic sequences, and when used alone, particularly for the V9 region, returns a large amount of unidentified OTUs due to its three-domain primers.

To prevent non-eukaryotic OTUs from artificially inflating this number, all unidentified OTUs were compared against the full SILVA database and any non-18S rRNA sequences were removed from the dataset. Some of the taxonomic levels added to our curated database, for example Hacrobia, do not reflect the current views of eukaryotic taxonomy from [16] or [60]; however, they were added in an effort to reduce the variability of taxonomic information present between major clades in the database and make summarizing taxonomic information (summarize_taxa.py) in QIIME easier. Even after use of these customized databases, unfortunately, significant amounts of unidentified OTUs remain. This further highlights the need for comprehensive eukaryotic microbe reference databases, and the vast number of eukaryotic microbes that remain to be described.

Our study aimed to provide a workflow for the detection and analysis of protists in sewage samples, with a focus on zoonotic and trichomonad taxa, based on high-throughput amplicon sequencing of existing 18S rRNA markers. As such, the method provides a snapshot of microbial eukaryotic diversity (living or dead) in sewage; we are unable to measure active vs. dormant microbes, nor assess what could be considered “residual” or “transitory” species. Additional complementary analyses will be needed to determine whether the detected species are metabolically active, and to examine the likely sources of these species. The 18S rRNA regions used in this study were limited in their ability to provide fine-scale taxonomic resolutions between species or strains of zoonotic taxa, particularly between closely related trichomonads; however, they can be used to track potential large-scale trends that may influence the distribution of zoonotic microbes in urban environments. Although detection of these biomarker taxa do not provide quantitative estimates of abundance, our method provides a jumping off point for future targeted study design and hypothesis testing to confirm the source, viability, and distribution of these pathogenic species.

## Electronic supplementary material

Online Resource 1 details of the DNA sources used in this study. Gives strain/isolate name, provider, geographical location and source (where known). References are provided for published isolates/strains not available through ATCC or other sources. (PDF 69 kb).

Online Resource 2 Multiple sequence alignments of Sanger sequence data in FASTA format generated from V4 region of 18S rRNA gene of 63 protist, vertebrate and fungus samples. (FASTA 34 kb).

Online Resource 3 Multiple sequence alignments of Sanger sequence data in FASTA format generated from V9 region of 18S rRNA gene of 62 protist, vertebrate and fungus samples. (FASTA 11 kb).

Online Resource 4 GenBank accession numbers and taxonomic string of the sequences added to our curated database. (TXT 2 kb).

Online Resource 5 results of OTU clustering and taxonomic assignment for the V4 Sanger sequences at both 97% and 98% identity. Includes the number and identity of sequences represented by each OTU, source of each representative sequence, the taxonomy and e-value assigned to each OTU using both the SILVA 111 reference database and our curated version, for each threshold. (PDF 168 kb).

Online Resource 6 results of OTU clustering and taxonomic assignment for the V9 Sanger sequences. Includes the number, number and identity of sequences represented by each OTU, source of each representative sequence, the taxonomy and e-value assigned to each OTU using both the SILVA 111 reference database and our curated version. As clustering at 97% and 98% produced identical results, only 98% is included here. (PDF 123 kb).

Online Resource 7 Rarefaction curves for protist only OTU tables rarefied from 60,000 to 600,000 sequences with the Phylogenetic Diversity metric. Error bars are standard deviation. Lines that do not extend all the way to the end of the x-axis indicate that at least one sample in that category does not have that many sequences. (A) V9 region and (B) V4 region. (PDF 211 kb).
