# Supplementary material for: An 18S rRNA Workflow for Characterizing Protists in Sewage, with a Focus on Zoonotic Trichomonads
Source: Microb Ecol. 2017 May 24;74(4):923–36. doi: 10.1007/s00248-017-0996-9 (PMC5653731; doi:10.1007/s00248-017-0996-9)
Supplement: Supplementary file 1 — details of the DNA sources used in this study. Gives strain/isolate name, provider, geographical location and source (where known). References are provided for published isolates/strains not available through ATCC or other sources. (PDF 69 kb). [file 248_2017_996_MOESM1_ESM.pdf]

An 18S rRNA workflow for characterizing protists in sewage, with a focus on zoonotic trichomonads; Microbial Ecology; Maritz, JM, Rogers, KH, Rock, TM, Liu N, Joseph, S. Land, KM, Carlton, JM*;
*corresponding author, Center for Genomics and Systems Biology, Department of Biology, New York University, jane.carlton@nyu.edu

Online Resource 1 details of the DNA sources used in this study. Gives strain/isolate name, provider, geographical location and source (where known).
References are provided for published isolates/strains not available through ATCC or other sources.

Genus and species	Strain or isolate	Provider	Geographical location (where known)	Source (where known)	Reference
Trichomonas vaginalis	G3	ATCC #PRA-98	Kent, England	Human	
Trichomonas vaginalis	C1:NIH	ATCC #30001	Maryland, USA	Human female	
Trichomonas vaginalis	TAIHS/03/HEPU/192	Carlton Lab	Northern Queensland, Australia	Human	1
Trichomonas vaginalis	TAIHS/03/HEPU/176	Carlton Lab	Northern Queensland, Australia	Human	1
Trichomonas vaginalis	DUR/03/FMUN/A19	Carlton Lab	Rural Kwazulu-Natal, SA	Human	1
Trichomonas vaginalis	DUR/03/FMUN/A71	Carlton Lab	Rural Kwazulu-Natal, SA	Human	1
Trichomonas vaginalis	DUR/03/FMUN/30	Carlton Lab	Rural Kwazulu-Natal, SA	Human	1
Trichomonas vaginalis	GOR/03/PNGIMR/23	Carlton Lab	Goroka, PPNG	Human	1
Trichomonas vaginalis	GOR/03/PNGIMR/21	Carlton Lab	Goroka, PPNG	Human	1
Trichomonas vaginalis	PMGH25	Carlton Lab	Port Moresby, PNG	Human	1
Trichomonas vaginalis	NYCF20	Carlton Lab	New York, NY	Human	1
Trichomonas vaginalis	NYCE32	Carlton Lab	Brooklyn, NY	Human	1
Trichomonas vaginalis	NYCC37	Carlton Lab	New York, NY	Human	1
Trichomonas vaginalis	NYCB20	Carlton Lab	Brooklyn, NY	Human	1
Trichomonas vaginalis	NYCD15	Carlton Lab	New York, NY	Human	1
Trichomonas vaginalis	NYCA04	Carlton Lab	Brooklyn, NY	Human	1
Trichomonas vaginalis	SD1	Carlton Lab	San Diego, CA	Human	1
Trichomonas vaginalis	SD7	Carlton Lab	San Diego, CA	Human	1
Trichomonas vaginalis	1080	Carlton Lab	New York, NY	Human	1
Trichomonas vaginalis	1035	Carlton Lab	Brooklyn, NY	Human	1
Trichomonas vaginalis	1031	Carlton Lab	New Lenox, IL	Human	1
Trichomonas vaginalis	CDC252	Carlton Lab	San Diego, CA	Human	1
Trichomonas vaginalis	CNDC188	Carlton Lab	Mexico City, Mexico	Human	1
Trichomonas vaginalis	CNDC217	Carlton Lab	Mexico City, Mexico	Human	1
Trichomonas vaginalis-like	12839	Kirkwood Land	California, USA	Band-tailed pigeon	Unpublished
Trichomonas vaginalis-like	12840	Kirkwood Land	California, USA	Band-tailed pigeon	Unpublished
Trichomonas vaginalis-like	12842	Kirkwood Land	California, USA	Band-tailed pigeon	Unpublished
Trichomonas vaginalis-like	12847	Kirkwood Land	California, USA	Band-tailed pigeon	Unpublished
Trichomonas vaginalis-like	12850	Kirkwood Land	California, USA	Band-tailed pigeon	Unpublished
Trichomonas vaginalis-like	12855	Kirkwood Land	California, USA	Band-tailed pigeon	Unpublished
Trichomonas vaginalis-like	12857	Kirkwood Land	California, USA	Band-tailed pigeon	Unpublished
Trichomonas vaginalis-like	13207	Kirkwood Land	California, USA	Eurasian collared dove	Unpublished
Trichomonas vaginalis-like	13211	Kirkwood Land	California, USA	Eurasian collared dove	Unpublished
Trichomonas vaginalis-like	13240	Kirkwood Land	California, USA	Eurasian collared dove	Unpublished
Trichomonas vaginalis-like	13255	Kirkwood Land	California, USA	Eurasian collared dove	Unpublished
Trichomonas gallinae	SL substrain of DP3	ATCC #30096	Washington DC, USA	Pigeon (*Columba livia*)	
Trichomonas gallinae	DP3 of Jones Barn (JB)	ATCC #30095	Pensylvania, USA	Wild squab	
Trichomonas gallinae	AG (Amherst)	ATCC #30002	Masachusettes, USA	Pigeon (*Columba livia*)	
Trichomonas gallinae	TG	ATCC #30230	Not provided	Pigeon (*Columba sp.*)	
Trichomonas tenax	Hs-4:NIH	ATCC #30207	Not provided	Human female	
Tetratrichomonas gallinarum	Leverett	ATCC #30249	Masachusettes, USA	Chicken (*Gallus sp.*)	
Tetratrichomonas gallinarum	TP-79	ATCC #30097	Maryland, USA	Domestic turkey	
Pentatrichomonas hominis	Hs-3:NIH	ATCC #30000	Korea	Human intestine	
Dientamoeba fragilis	Genotype 1	Graham Clark	Not provided	Human feces	2
Tritrichomonas foetus	KV-1	ATCC #30924	Czech Republic	*Bos taurus*	
Trichomitus batrachorum	G43	ATCC #30068	Bronx Zoo, NY USA	Snake (*Vipera russell*)	
Monocercomonas colubrorum	W-578-73	ATCC #30226	Ontario, Canada	*Iguana iguana*	
Monotrichomonas carabina	QBSA-1	ATCC #50700	Not provided	Free-living	
Ditrichomonas honigbergii	DR	ATCC #50322	Dominician Republic	Free-living, lake sediment	
Cryptosporidium parvum	Iowa	ATCC #PRA-67D	Not provided	Animal feces	
Toxoplasma gondii	RH	ATCC #50174D	Ohio, USA	Human male	

Blastocystis hominis	BT1	ATCC #50608D	Not provided	Human feces	
Giardia intestinalis	Portland-1	ATCC #30888D	Portland, USA	Human female	
Entamoeba histolytica	HM-1:IMSS	ATCC #30459	Mexico City, Mexico	Human male	
Entamoeba invadens	IP-1	ATCC #30994	Florida, USA	Snake (*Natrix cyclopion*)	
Saccharomyces cerevisiae	SK1	ATCC #204722	Not provided	*Saccharomyces cerevisiae*	
Homo sapiens	NA	Novagen 69237	Not provided	Human	
Rattus sp.	Not provided	BioChain D1434999-G02	Not provided	Female rat	
Gallus gallus	Not provided	BioChain D1C34999-G02	Not provided	Female chicken	
Canis lupus familiaris	Not provided	BioChain D1734999-G02	Not provided	Female dog	
Equus caballus	Not provided	BioChain D1O34999-G02	Not provided	Female horse	

References

Conrad MD, Gorman AW, Schillinger JA, Fiori PL, Arroyo R, Malla N, Dubey ML, Gonzalez J, Blank S, Secor WE et al:
Extensive Genetic Diversity, Unique Population Structure and Evidence of Genetic Exchange in the Sexually Transmitted Parasite Trichomonas vaginalis.
1 Plos Neglect Trop D 2012, 6(3).
2 Johnson JA & Clark CG: Cryptic diversity in Dientamoeba fragilis. J Clin Microbiol 2000, 38(12).
