# Supplementary material for: An 18S rRNA Workflow for Characterizing Protists in Sewage, with a Focus on Zoonotic Trichomonads
Source: Microb Ecol. 2017 May 24;74(4):923–36. doi: 10.1007/s00248-017-0996-9 (PMC5653731; doi:10.1007/s00248-017-0996-9)
Supplement: Supplementary file 5 — results of OTU clustering and taxonomic assignment for the V4 Sanger sequences at both 97% and 98% identity. Includes the number and identity of sequences represented by each OTU, source of each representative sequence, the taxonomy and e-value assigned to each OTU using both the SILVA 111 reference database and our curated version, for each threshold. (PDF 168 kb). [file 248_2017_996_MOESM5_ESM.pdf]

An 18S rRNA workflow for characterizing protists in sewage, with a focus on zoonotic trichomonads;

Microbial Ecology;

Maritz, JM, Rogers, KH, Rock, TM, Liu N, Joseph, S. Land, KM, Carlton, JM*;

*corresponding author, Center for Genomics and Systems Biology, Department of Biology, New York University, jane.carlton@nyu.edu

Online Resource 5 number and identity of Sanger sequences represented by each OTU clustered at 97% V4 region

OTU number	Sequences included		
1	Trichomonas_vaginalis_THAIS192	Trichomonas_vaginalis_THAIS176	Trichomonas_vaginalis_SD7
	Trichomonas_vaginalis_SD1	Trichomonas_vaginalis_SA-A71	Trichomonas_vaginalis_SA-A19
	Trichomonas_vaginalis_SA30	Trichomonas_vaginalis_PMGH25	Trichomonas_vaginalis_NYCF20
	Trichomonas_vaginalis_NYCE32	Trichomonas_vaginalis_NYCD15	Trichomonas_vaginalis_NYCC37
	Trichomonas_vaginalis_NYCB20	Trichomonas_vaginalis_NYCA04	Trichomonas_vaginalis-like_13255
	Trichomonas_vaginalis-like_13240	Trichomonas_vaginalis-like_13211	Trichomonas_vaginalis-like_13207
	Trichomonas_vaginalis-like_12857	Trichomonas_vaginalis-like_12855	Trichomonas_vaginalis-like_12850
	Trichomonas_vaginalis-like_12847	Trichomonas_vaginalis-like_12842	Trichomonas_vaginalis-like_12840
	Trichomonas_vaginalis-like_12839	Trichomonas_vaginalis_GOR23	Trichomonas_vaginalis_GOR21
	Trichomonas_vaginalis_G3	Trichomonas_vaginalis_CNDC217	Trichomonas_vaginalis_CNDC188
	Trichomonas_vaginalis_CDC252	Trichomonas_vaginalis_C1:NIH	Trichomonas_vaginalis_1080_
	Trichomonas_vaginalis_1035	Trichomonas_vaginalis_1031	Trichomonas_tenax_Hs-4:NIH
	Trichomonas_gallinae_TG	Trichomonas_gallinae_SL	Trichomonas_gallinae_DP3
	Trichomonas_gallinae_AG	Tetratrichomonas_gallinarum_TP-79	
2	Rattus_sp.	Homo_sapiens	Equus_caballus
	Canis_lupus_familiaris		
3	Monotrichomonas_carabina_QBSA-1		
4	Pentatrichomonas_hominis_Hs-3:NIH		
5	Tetratrichomonas_gallinarum_Leverett		
6	Saccharomyces_cerevisiae_SK1		
7	Ditrichomonas_honigbergii_DR		
8	Cryptospordium_parvum_Iowa		
9	Blastocystis_hominis_BT1		
10	Monocercomonas_colubrorum_W-578-73		
11	Entamoeba_invadens_IP-1	AF149905_Entamoeba_invadens	
12	Entamoeba_histolytica_HM-1:IMSS		
13	M54878_Giardia_intestinalis_Portland-1		
14	Trichomitus_batrachorum_G43		
15	Toxoplasma_gondii_RH		
16	Gallus_gallus		
17	Tritrichomonas_foetus_KV-1		
18	U37461_Dientamoeba_fragilis_Bi/PA		
19	Dientamoeba_fragilis_Genotype1		

Online Resource 5 taxonomic assignment for the V4 Sanger sequences at 97%

OTU number	Number of sequences assigned	Source of OTU representative sequence	Taxonomy assigned, SILVA 111 db (evalue)	Taxonomy assigned, curated db (evalue)
1	41	*Trichomonas vaginalis THAIS192*	*Trichomonas vaginalis* (9E-155)	*Trichomonas vaginalis* (7E-156)
2	4	*Rattus sp.*	*Homo sapiens* (0)	*Homo sapiens* (0)
3	1	*Monotrichomonas carabina* QBSA-1	*Pseudotrichomonas sp.* (1E-95)	*Monotrichomonas carabina* (5E-157)
4	1	*Pentatrichomonas hominis* Hs-3:NIH	*Pentatrichomonas hominis* (1E-153)	*Pentatrichomonas hominis* (1E-154)
5	1	*Tetratrichomonas gallinarum* Leverett	*Tetratrichomonas gallinarum* (3E-142)	*Tetratrichomonas gallinarum* (3E-143)
6	1	*Saccharomyces cerevisiae*	*Saccharomyces cerevisiae* (0)	*Saccharomyces cerevisiae* (0)
7	1	*Ditrichomonas honigbergii* DR	*Honigbergiellidae sp.* (3E-117)	*Ditrichomonas honigbergii* (1E-145)
8	1	*Cryptospordium parvum* Iowa	*Cryptosporidium parvum* (7E-122)	*Cryptosporidium parvum* (6E-123)
9	1	*Blastocystis hominis* BT1	*Blastocystis sp.* (0)	*Blastocystis sp.* (0)
10	1	*Monocercomonas colubrorum* W-578-73	*Monocercomonas colubrorum* (2E-130)	*Monocercomonas colubrorum* (2E-131)
11	2	*Entamoeba invadens* IP-1	No blast hit (None)	*Entamoeba invadens* (0)
12	1	*Entamoeba histolytica* HM-1:IMSS	No blast hit (None)	*Entamoeba histolytica* (0)
13	1	M54878 *Giardia intestinalis* Portland-1	*Giardia intestinalis* (2E-50)	*Giardia intestinalis* (2E-51)
14	1	*Trichomitus batrachorum* G43	*Trichomitus batrachorum* (5E-150)	*Trichomitus batrachorum* (4E-151)
15	1	*Toxoplasma gondii* RH	*Toxoplasma gondii* (0)	*Toxoplasma gondii* (0)
16	1	*Gallus gallus*	*Gallus gallus* (0)	*Gallus gallus* (0)
17	1	*Tritrichomonas foetus* KV-1	*Tritrichomonas suis* (1E-150)*	*Tritrichomonas suis* (1E-151)
18	1	U37461 *Dientamoeba fragilis* Bi/PA	*Dientamoeba fragilis* (6E-54)	*Dientamoeba fragilis* (5E-55)
19	1	*Dientamoeba fragilis* Genotype1	*Dientamoeba fragilis* (7E-57)	*Dientamoeba fragilis* (6E-58)

**Tritrichomonas foetus* and *Tritrichomonas suis* are considered the same species

Online Resource 5 number and identity of Sanger sequences represented by each OTU clustered at 98% V4 region

<table>
<tr><th>OTU number</th><th colspan="3">Sequences included</th></tr>
<tr><td rowspan="8">1</td><td>Trichomonas_vaginalis_THAIS192</td><td>Trichomonas_vaginalis_THAIS176</td><td>Trichomonas_vaginalis_SD7</td></tr>
<tr><td>Trichomonas_vaginalis_SD1</td><td>Trichomonas_vaginalis_SA-A71</td><td>Trichomonas_vaginalis_SA-A19</td></tr>
<tr><td>Trichomonas_vaginalis_SA30</td><td>Trichomonas_vaginalis_PMGH25</td><td>Trichomonas_vaginalis_NYCF20</td></tr>
<tr><td>Trichomonas_vaginalis_NYCE32</td><td>Trichomonas_vaginalis_NYCD15</td><td>Trichomonas_vaginalis_NYCC37</td></tr>
<tr><td>Trichomonas_vaginalis_NYCB20</td><td>Trichomonas_vaginalis_NYCA04</td><td>Trichomonas_vaginalis_GOR23</td></tr>
<tr><td>Trichomonas_vaginalis_GOR21</td><td>Trichomonas_vaginalis_G3</td><td>Trichomonas_vaginalis_CNDC217</td></tr>
<tr><td>Trichomonas_vaginalis_CNDC188</td><td>Trichomonas_vaginalis_CDC252</td><td>Trichomonas_vaginalis_1080_</td></tr>
<tr><td>Trichomonas_vaginalis_1035</td><td>Trichomonas_vaginalis_1031</td><td></td></tr>
<tr><td rowspan="6">2</td><td>Trichomonas_vaginalis-like_13255</td><td>Trichomonas_vaginalis-like_13240</td><td>Trichomonas_vaginalis-like_13211</td></tr>
<tr><td>Trichomonas_vaginalis-like_12857</td><td>Trichomonas_vaginalis-like_12855</td><td>Trichomonas_vaginalis-like_13207</td></tr>
<tr><td>Trichomonas_vaginalis-like_12850</td><td>Trichomonas_vaginalis-like_12847</td><td>Trichomonas_vaginalis-like_12842</td></tr>
<tr><td>Trichomonas_vaginalis-like_12840</td><td>Trichomonas_vaginalis-like_12839</td><td>Trichomonas_vaginalis_C1:NIH</td></tr>
<tr><td>Trichomonas_tenax_Hs-4:NIH</td><td>Trichomonas_gallinae_TG</td><td>Trichomonas_gallinae_DP3</td></tr>
<tr><td>Trichomonas_gallinae_SL</td><td>Trichomonas_gallinae_AG</td><td>Tetratrichomonas_gallinarum_TP-79</td></tr>
<tr><td rowspan="2">3</td><td>Rattus_sp.</td><td>Homo_sapiens</td><td>Equus_caballus</td></tr>
<tr><td>Canis_lupus_familiaris</td><td></td><td></td></tr>
<tr><td>4</td><td>Monotrichomonas_carabina_QBSA-1</td><td></td><td></td></tr>
<tr><td>5</td><td>Pentatrichomonas_hominis_Hs-3:NIH</td><td></td><td></td></tr>
<tr><td>6</td><td>Tetratrichomonas_gallinarum_Leverett</td><td></td><td></td></tr>
<tr><td>7</td><td>Saccharomyces_cerevisiae_SK1</td><td></td><td></td></tr>
<tr><td>8</td><td>Ditrichomonas_honigbergii_DR</td><td></td><td></td></tr>
<tr><td>9</td><td>Cryptospordium_parvum_Iowa</td><td></td><td></td></tr>
<tr><td>10</td><td>Blastocystis_hominis_BT1</td><td></td><td></td></tr>
<tr><td>11</td><td>Monocercomonas_colubrorum_W-578-73</td><td></td><td></td></tr>
<tr><td>12</td><td>Entamoeba_invadens_IP-1</td><td>AF149905_Entamoeba_invadens</td><td></td></tr>
<tr><td>13</td><td>Entamoeba_histolytica_HM-1:IMSS</td><td></td><td></td></tr>
<tr><td>14</td><td>M54878_Giardia_intestinalis_Portland-1</td><td></td><td></td></tr>
<tr><td>15</td><td>Trichomitus_batrachorum_G43</td><td></td><td></td></tr>
<tr><td>16</td><td>Toxoplasma_gondii_RH</td><td></td><td></td></tr>
<tr><td>17</td><td>Gallus_gallus</td><td></td><td></td></tr>
<tr><td>18</td><td>Tritrichomonas_foetus_KV-1</td><td></td><td></td></tr>
<tr><td>19</td><td>U37461_Dientamoeba_fragilis_Bi/PA</td><td></td><td></td></tr>
<tr><td>20</td><td>Dientamoeba_fragilis_Genotype1</td><td></td><td></td></tr>
</table>

Online Resource 5 taxonomic assignment for the V4 Sanger sequences at 98%

OTU number	Number of sequences assigned	Source of OTU representative sequence	Taxonomy assigned, SILVA 111 db (evalue)	Taxonomy assigned, curated db (evalue)
1	23	*Trichomonas vaginalis THAIS192*	*Trichomonas vaginalis* (9E-155)	*Trichomonas vaginalis* (7E-156)
2	18	Trichomonas vaginalis-like 13255	*Trichomonas gallinae* (9E-155)	*Trichomonas gallinae* (7E-156)
3	4	*Rattus sp.*	*Homo sapiens* (0)	*Homo sapiens (0)*
4	1	*Monotrichomonas carabina* QBSA-1	*Pseudotrichomonas sp.* (1E-95)	*Monotrichomonas carabina* (5E-157)
5	1	*Pentatrichomonas hominis* Hs-3:NIH	*Pentatrichomonas hominis* (1E-153)	*Pentatrichomonas hominis* (1E-154)
6	1	*Tetratrichomonas gallinarum* Leverett	*Tetratrichomonas gallinarum* (3E-142)	*Tetratrichomonas gallinarum* (3E-143)
7	1	*Saccharomyces cerevisiae*	*Saccharomyces cerevisiae* (0)	*Saccharomyces cerevisiae* (0)
8	1	*Ditrichomonas honigbergii* DR	*Honigbergiellidae sp.* (3E-117)	*Ditrichomonas honigbergii* (1E-145)
9	1	*Cryptospordium parvum* Iowa	*Cryptosporidium parvum* (7E-122)	*Cryptosporidium parvum* (6E-123)
10	1	*Blastocystis hominis* BT1	*Blastocystis sp.* (0)	*Blastocystis sp.* (0)
11	1	*Monocercomonas colubrorum* W-578-73	*Monocercomonas colubrorum* (2E-130)	*Monocercomonas colubrorum* (2E-131)
12	2	*Entamoeba invadens* IP-1	No blast hit (None)	*Entamoeba invadens* (0)
13	1	*Entamoeba histolytica* HM-1:IMSS	No blast hit (None)	*Entamoeba histolytica* (0)
14	1	M54878 *Giardia intestinalis* Portland-1	*Giardia intestinalis* (2E-50)	*Giardia intestinalis* (2E-51)
15	1	*Trichomitus batrachorum* G43	*Trichomitus batrachorum* (5E-150)	*Trichomitus batrachorum* (4E-151)
16	1	*Toxoplasma gondii* RH	*Toxoplasma gondii* (0)	*Toxoplasma gondii* (0)
17	1	*Gallus gallus*	*Gallus gallus* (0)	*Gallus gallus* (0)
18	1	*Tritrichomonas foetus* KV-1	*Tritrichomonas suis* (1E-150)*	*Tritrichomonas suis* (1E-151)*
19	1	U37461 *Dientamoeba fragilis* Bi/PA	*Dientamoeba fragilis* (6E-54)	*Dientamoeba fragilis* (5E-55)
20	1	*Dientamoeba fragilis* Genotype1	*Dientamoeba fragilis* (7E-57)	*Dientamoeba fragilis* (6E-58)

*****Tritrichomonas foetus* and *Tritrichomonas suis* are considered the same species
