# Supplementary material for: An 18S rRNA Workflow for Characterizing Protists in Sewage, with a Focus on Zoonotic Trichomonads
Source: Microb Ecol. 2017 May 24;74(4):923–36. doi: 10.1007/s00248-017-0996-9 (PMC5653731; doi:10.1007/s00248-017-0996-9)
Supplement: Supplementary file 6 — results of OTU clustering and taxonomic assignment for the V9 Sanger sequences. Includes the number, number and identity of sequences represented by each OTU, source of each representative sequence, the taxonomy and e-value assigned to each OTU using both the SILVA 111 reference database and our curated version. As clustering at 97% and 98% produced identical results, only 98% is included here. (PDF 123 kb). [file 248_2017_996_MOESM6_ESM.pdf]

An 18S rRNA workflow for characterizing protists in sewage, with a focus on zoonotic trichomonads;

Microbial Ecology;

Maritz, JM, Rogers, KH, Rock, TM, Liu N, Joseph, S. Land, KM, Carlton, JM*;

*corresponding author, Center for Genomics and Systems Biology, Department of Biology, New York University, jane.carlton@nyu.edu

Online Resource 6 number and identity of sequences represented by each OTU clustered at 98%.
As clustering at 97% and 98% produced identical results, only 98% is included here.

OTU number	Sequences included		
1	Trichomonas_vaginalis_THAIS192	Trichomonas_vaginalis_THAIS176	Trichomonas_vaginalis_SD7
	Trichomonas_vaginalis_SD1	Trichomonas_vaginalis_SA-A71	Trichomonas_vaginalis_SA-A19
	Trichomonas_vaginalis_SA30	Trichomonas_vaginalis_PMGH25	Trichomonas_vaginalis_NYCF20
	Trichomonas_vaginalis_NYCE32	Trichomonas_vaginalis_NYCD15	Trichomonas_vaginalis_NYCC37
	Trichomonas_vaginalis_NYCB20	Trichomonas_vaginalis_NYCA04	Trichomonas_vaginalis-like_13255
	Trichomonas_vaginalis-like_13240	Trichomonas_vaginalis-like_13211	Trichomonas_vaginalis-like_13207
	Trichomonas_vaginalis-like_12857	Trichomonas_vaginalis-like_12855	Trichomonas_vaginalis-like_12850
	Trichomonas_vaginalis-like_12847	Trichomonas_vaginalis-like_12842	Trichomonas_vaginalis-like_12840
	Trichomonas_vaginalis-like_12839	Trichomonas_vaginalis_GOR23	Trichomonas_vaginalis_GOR21
	Trichomonas_vaginalis_G3	Trichomonas_vaginalis_CNDC217	Trichomonas_vaginalis_CNDC188
	Trichomonas_vaginalis_CDC252	Trichomonas_vaginalis_C1:NIH	Trichomonas_vaginalis_1080_
	Trichomonas_vaginalis_1035	Trichomonas_vaginalis_1031	Trichomonas_tenax_Hs-4:NIH
	Trichomonas_gallinae_TG	Trichomonas_gallinae_SL	Trichomonas_gallinae_DP3
	Trichomonas_gallinae_AG	Tetratrichomonas_gallinarum_TP-79	
2	Rattus_sp.	Homo_sapiens	Equus_caballus
	Canis_lupus_familiaris		
3	M54878_Giardia_intestinalis_Portland-1	Giardia_intestinalis_Portland-1	
4	Toxoplasma_gondii_RH		
5	Trichomitus_batrachorum_G43		
6	Tetratrichomonas_gallinarum_Leverett		
7	Blastocystis_hominis_BT1		
8	AF149905_Entamoeba_invadens		
9	Ditrichomonas_honigbergii_DR		
10	Cryptosporidium_parvum_Iowa		
11	Saccharomyces_cerevisiae_SK1		
12	Pentatrichomonas_hominis_Hs-3:NIH		
13	U37461_Dientamoeba_fragilis_Bi/PA		
14	Tritrichomonas_foetus_KV-1		
15	Gallus_gallus		
16	Entamoeba_histolytica_HM-1:IMSS		
17	Monotrichomonas_carabina_QBSA-1		
18	Monocercomonas_colubrorum_W-578-73		

Online Resource 6 taxonomic assignment for the V4 Sanger sequences at 98%. As clustering at 97% and 98% produced identical results, only 98% is included here.

OTU number	Number of sequences assigned	Source of OTU representative sequence	Taxonomy assigned SILVA 111 db (evalue)	Taxonomy assigned curated db (evalue)
1	41	*Trichomonas vaginalis* THAIS192	*Trichomonas tenax* (2E-51)	*Trichomonas sp.* (2E-52)
2	4	*Rattus sp.*	*Mus musculus* (8E-70)	*Mus musculus* (7E-71)
3	2	*Giardia intestinalis* Portland-1	*Giardia intestinalis* (3E-47)	*Giardia intestinalis* (2E-48)
4	1	*Toxoplasma gondii* RH	*Toxoplasma gondii* (1E-62)	*Toxoplasma gondii* (9E-64)
5	1	*Trichomitus batrachorum* G43	*Trichomitus batrachorum* (1E-34)	*Trichomitus batrachorum* (8E-36)
6	1	*Tetratrichomonas gallinarum* Leverett	*Tetratrichomonas sp.* (5E-49)	*Tetratrichomonas sp.* (4E-50)
7	1	*Blastocystis hominis* BT1	*Blastocystis sp.* (5E-68)	*Blastocystis sp.* (4E-69)
8	1	AF149905 *Entamoeba invadens*	*Entamoeba suis* (1.00E-15)	*Entamoeba invadens* (1E-56)
9	1	*Ditrichomonas honigbergii* DR	AB183887 *uncultured parabasalid eukaryote* (1E-40)	*Ditrichomonas honigbergii* (3E-51)
10	1	*Cryptosporidium parvum* Iowa	*Cryptosporidium wrairi* (3E-60)	*Cryptosporidium wrairi* (2E-61)
11	1	Saccharomyces cerevisiae	*Saccharomyces cerevisiae* (8E-67)	*Saccharomyces cerevisiae* (7E-68)
12	1	*Pentatrichomonas hominis* Hs-3:NIH	*Pentatrichomonas hominis* (3E-50)	*Pentatrichomonas hominis* (3E-51)
13	1	U37461 *Dientamoeba fragilis* Bi/PA	*Dientamoeba fragilis* (7E-39)	*Dientamoeba fragilis* (6E-40)
14	1	*Tritrichomonas foetus* KV-1	*Tritrichomonas suis* (3E-50)*	*Tritrichomonas suis* (3E-51)*
15	1	Gallus gallus	*Gallus gallus* (3E-69)	*Gallus gallus* (3E-70)
16	1	*Entamoeba histolytica* HM-1:IMSS	No blast hit (None)	*Entamoeba dispar* (4E-50)
17	1	*Monotrichomonas carabina* QBSA-1	*Tritrichomonas suis* (2E-36)	*Monotrichomonas carabina* (4E-50)
18	1	*Monocercomonas colubrorum* W-578-73	*Pseudotrichomonas keilini* (2E-29)	*Ditrichomonas honigbergii* (5E-31)

*****_Tritrichomonas foetus_ and *Tritrichomonas suis* are considered the same species
