# Supplementary material for: An 18S rRNA Workflow for Characterizing Protists in Sewage, with a Focus on Zoonotic Trichomonads
Source: Microb Ecol. 2017 May 24;74(4):923–36. doi: 10.1007/s00248-017-0996-9 (PMC5653731; doi:10.1007/s00248-017-0996-9)
Supplement: Supplementary file 7 — Rarefaction curves for protist only OTU tables rarefied from 60,000 to 600,000 sequences with the Phylogenetic Diversity metric. Error bars are standard deviation. Lines that do not extend all the way to the end of the x-axis indicate that at least one sample in that category does not have that many sequences. (A) V9 region and (B) V4 region. (PDF 211 kb). [file 248_2017_996_MOESM7_ESM.pdf]

An 18S rRNA workflow for characterizing protists in sewage, with a focus on zoonotic trichomonads;
Microbial Ecology;
Maritz, JM, Rogers, KH, Rock, TM, Liu N, Joseph, S. Land, KM, Carlton, JM*;
*corresponding author, Center for Genomics and Systems Biology, Department of Biology, New York University, jane.carlton@nyu.edu

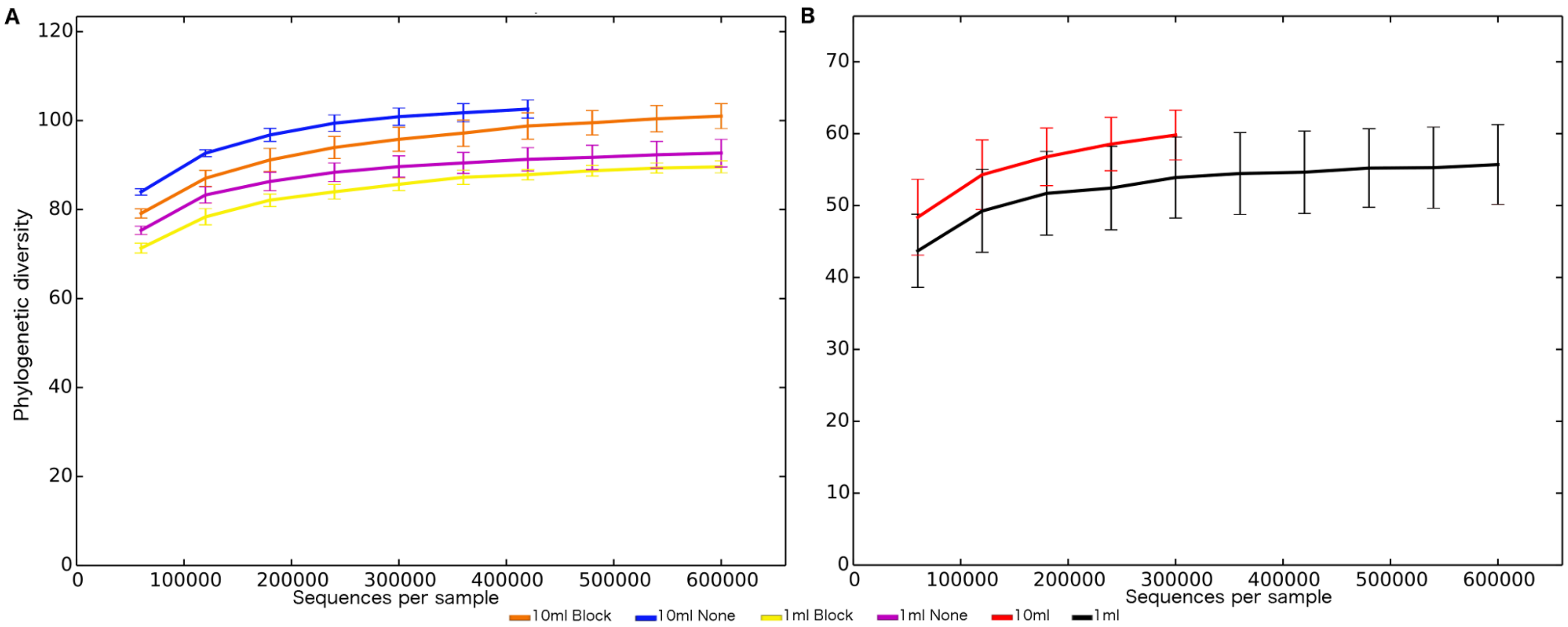

Online Resource 7 Rarefaction curves for protist only OTU tables rarefied from 60,000 to 600,000 sequences with the Phylogenetic Diversity metric. Error bars are standard deviation. Lines that do not extend all the way to the end of the x-axis indicate that at least one sample in that category does not have that many sequences. (A) V9 region and (B) V4 region.
